# Gas Chromatography Chemometrics of Volatile Analytes in Mexican Mezcals: Fingerprinting Ancestral and Artisanal Manufacturing Processes, Geographical Origins, and Agave Species

**DOI:** 10.3390/foods15152711

**Published:** 2026-07-31

**Authors:** Rosa López-Aguilar, Emanuel Hernández-Núñez, Germán Giácoman-Vallejos, Avel González-Sánchez, Arturo Hernández-Montes, José Enrique Herbert-Pucheta

**Affiliations:** 1 Instituto Tecnológico de Zitácuaro, Tecnológico Nacional de México, Área Agroalimentaria, Zitácuaro 61534, Michoacán, Mexico; 2 Departamento de Ingeniería Agroindustrial, Universidad Autónoma Chapingo, Km. 38.5 Carretera México, Texcoco 56230, Estado de México, Mexico; sensoarturo@gmail.com; 3 Departamento de Posgrado e Investigación, Instituto Tecnológico Superior de Calkiní, Avenida Ah Canul S/N por Carretera Federal, Calkiní 24930, Campeche, Mexico; ehernandez@itescam.edu.mx; 4 Facultad de Ingeniería, Universidad Autónoma de Yucatán, Av. Industrias No Contaminantes por Anillo Periférico Norte S/N, Cordemex, Mérida 97000, Yucatán, Mexico; giacoman@correo.uady.mx (G.G.-V.); avel.gonzalez@correo.uady.mx (A.G.-S.); 5 Centro Interdisciplinario de Investigación Para el Desarrollo Integral Regional Unidad Durango, Instituto Politécnico Nacional, Calle Sigma 119, Colonia 20 de Noviembre II, Durango 34220, Durango, Mexico

**Keywords:** gas chromatography chemometrics, ancestral and artisanal mezcal, Oaxaca mezcal, San Luis Potosí Mezcal, GC-MS-MSA

## Abstract

In this study, we developed a chemometrics approach for fingerprinting and identifying discriminant metabolites in Mexican mezcals. The approach was based on direct immersion solid-phase micro-extraction (DI-SPME) and gas chromatography–mass spectrometry (GC-MS). We used multivariate statistical analysis (MSA) to discriminate between ancestral and artisanal manufacturing processes, as well as between different geographical origins and agave species. The designed GC-MS-MSA separation method, labelled “Method 1”, enabled the unequivocal identification of twelve marker compounds that were confirmed with Electronic Impact Mass Spectrometry (EI-MS) mass-profile analyses, comprising a pairwise comparison between experimental *m*/*z* patterns and data obtained from the National Institute of Standards and Technology (NIST) MS library. Furthermore, to demonstrate the correlation between the reference and experimental mass spectra, Pearson correlation coefficients and their standard deviations were calculated. In all cases, Pearson’s correlation coefficient presented values with ranges between 0.96 and 0.99, indicating a high match between both spectra, whilst standard deviation values from 0.29 to 4.69 provided the data consistency. Supervised MSA of mezcals’ Method 1 GC cohorts (16 samples) are able to distinguish between different geographical origins (San Luis Potosí, Sierra sur Oaxaca and Valles Centrales, Oaxaca, R^2^[Y] = 0.79/Q^2^ = 0.79), agave species (*Agave potatorum*, *Agave angustifolia*, *Agave karwinskii*, *Agave salmiana*, *Agave marmorata*, and *Agave americana* ‘Oaxacensis’, R^2^[Y] = 0.79/Q^2^ = 0.79), and manufacturing processes (ancestral vs. artisanal, R^2^[Y] = 0.87/Q^2^ = 0.77).

## 1. Introduction

The health and safety of spirit consumers throughout México is seriously at risk, as around 30 to 50% of alcoholic beverages in the country are either illegal or of clandestine origin [1]. Consequently, the use of analytical methods which are able to trace metabolic signatures related to authenticity, variety and/or geographical origin is unambiguously justified within the framework of local public health. Mezcal production includes more than 50 agave species, with roughly 22 main agave species representing the total spirit domestic market; *Agave angustifolia* (Agave Espadín) is the most common and representative variety in México [2]. Mezcal is produced by distillation of previously cooked “piñas’” (agave’s head) to obtain fermented juice [3,4]. Mezcals’ volatile metabolome strongly depends on the following factors: (i) agave species; (ii) geographical origins; (iii) climate change impact; (iv) artisanal or ancestral manufacturing processes [5]. Differences between both manufacturing processes rely on the specific agave-cooking, must-fermentation and liqueur-distillation [6,7] practices. Agave species provide specific terpene profiles, which are responsible for the herbal aromas coming from terpinene, terpinol and their derivates [8]; they also produce flowery notes [9]. Traditional agave roasting is a slow cooking process carried out in earthen pit ovens, fueled with wood and lined rocks, that might produce furans and volatile organic compounds (VOCs) related to smoky or ashy aromas that are retained by agaves, such as volatile phenols produced by thermal degradation of lignin [10,11,12]. Must fermentation typically produces higher quantities of alcohols and ethyl esters related to fruity and floral aromatic notes, as well as the organic acids linked to cheesy aromas [8,13].

Targeted and untargeted NMR-based metabolomics analyses of non-volatile mezcal species have been recently reported [5]. Complementarily, VOC-targeted analyses with sample derivatization schemes conducted prior to analysis—such as solid-phase microextraction (SPME) or liquid–liquid extraction for hyphenated gas chromatography–mass spectrometry (GC-MS) analysis—has been recently proposed [14] to obtain the volatile metabolomes of mezcals.

Food integrity control, guaranteeing the safety, quality, authenticity, and traceability of consumables, is of central importance for consumer protection, inspection, and custom agencies. Worldwide control agencies are constantly promoting the use of analytical methods for detecting fraudulent practices such as counterfeited commodities, including spirits. Alcoholic beverages are especially prone to being adulterated since they can be easily mixed with a number of cheaper liquids [15]. In recent years, standard analytical reference methods are transitioning towards omics techniques, combining either separation and/or spectroscopic–spectrometric schemes. However, most approaches comprise targeted screening strategies. Recently, due to the implementation of robust multivariate statistical analyses in metabolomics [16], counterfeiting techniques are focused on recording unique sample fingerprints with non-targeted metabolomics approaches. Such holistic methods (related to food integrity control) can produce broad or narrow analytical signatures, depending on the capacity of the technique (these produce low- or high-resolution fingerprints, respectively). Typically, broad analytical fingerprints are obtained with benchtop instrumentation such as ultraviolet–visible (UV-VIS), Raman or near-infrared (NIR) approaches, whilst narrow fingerprints for elucidating complex chemical diversities are obtained by means of coupled chromatography with mass spectrometry (and, more recently, nuclear magnetic resonance spectroscopy) [17]. In alcoholic beverages, broad and narrow analytical fingerprints have been used to trace brand and generic authentication, respectively; for instance, they have been used to differentiate rums manufactured from sugar cane juice from those made from sugar cane molasses [18], and for the assessment of both quality and authenticity of Scotch Whisky. These assessments are based on a non-targeted fingerprinting approach using gas chromatography coupled with tandem high-resolution mass spectrometry; these steps are followed by multidimensional chemometrics processing [19]. Exciting examples of GC-MS foodomics applications have used coffee [20], green tea [21], wine food systems [22] and honey [23]. Regarding GC-MS chemometrics applied in spirit authentication, an excellent recent study proposes a GC-MS approach combined with linear discriminant analysis for distinguishing between Italian “grappa” from wine, grain, apple and pear spirits, as well as adulterations of suspicious samples [24]. To the best of our knowledge, GC-MS non-targeted and targeted metabolomics and chemometrics studies have not been proposed so far for fingerprinting or identifying discriminant metabolites in mezcals. The present work describes the implementation of a gas chromatography separation method for obtaining high-resolution chromatograms that can be used as GC data matrix for fingerprinting mezcal manufacturing processes, geographical origins and agave species with supervised multivariate statistical analysis. Twelve discriminant metabolites were identified from supervised partial least squares discriminant analysis (PLS-DA) loading plots, which were confirmed with their EI-MS profiles reported in public repositories. The aforementioned features are responsible for the discriminations between ancestral and artisanal manufacturing processes, geographical origins (among three counties in two Mexican States) and six different agave species.

## 2. Materials and Methods

### 2.1. Mezcal Sampling

**Figure 1:**
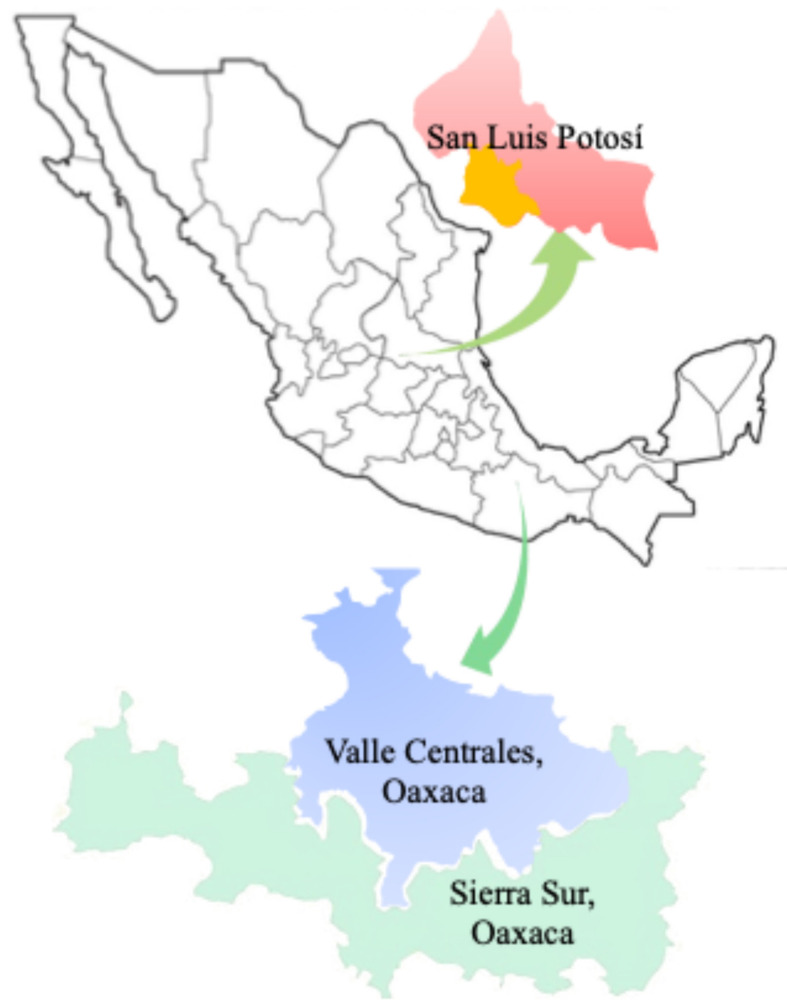
Map of México, indicating the States of San Luis Potosí (red) and Oaxaca, containing the regions of Valles Centrales (blue) and Sierra Sur (green). All samples reported in Table 1 were obtained from these regions.

The mezcal samples (Table 1) were sourced from manufacturers from the States of Oaxaca (Valles Centrales and Sierra Sur regions) and San Luis Potosí, México (22°9′4″ N, 100°58′34″ W). Both Valles Centrales (17°4′59.88″ N, 96°45′0″ W) and Sierra Sur (19°42′0″ N, 96°52′0″ W) regions in Oaxaca are well known for their mezcal production (Figure 1). The *Agave potatorum*, *Agave angustifolia*, *Agave karwinskii*, *Agave salmiana*, *Agave marmorata*, and *Agave americana* ‘Oaxacensis’ species were used to manufacture the herein-presented mezcal samples. Two processes are represented in their manufacture: artisanal and ancestral. The experimental units for this study were mezcal bottles with volumes of 250 mL per unit.

The samples were bottled by each producer in glass containers, labeled with the following information (required by the Mexican Norm NOM-070-SCFI-2016): category of the manufacturing process (artisanal or ancestral), class (young/aged mezcal), agave percentage (100% agave), agave name (example: espadín), net content (250 or 750 mL), alcohol by volume percentage (between 35 to 55%ABV), brand (example: Cuish^®^), producer information (tax information), Protected Designation of Origin [PDO], batch number, caution statements (responsible consumption), official Hologram of the Mexican Council of Mezcal’s Quality Regulation (Consejo Mexicano Regulador de la Calidad del Mezcal, COMERCAM in Spanish) and tax stamp from the Mexican Internal Revenue Service Bureau (Servicio de Administración Tributaria, SAT in Spanish). These details were required in determining the quality and authenticity certification for all samples listed in Table 1. All mezcals samples were stored at 4 °C until analysis.

**Figure 2:**
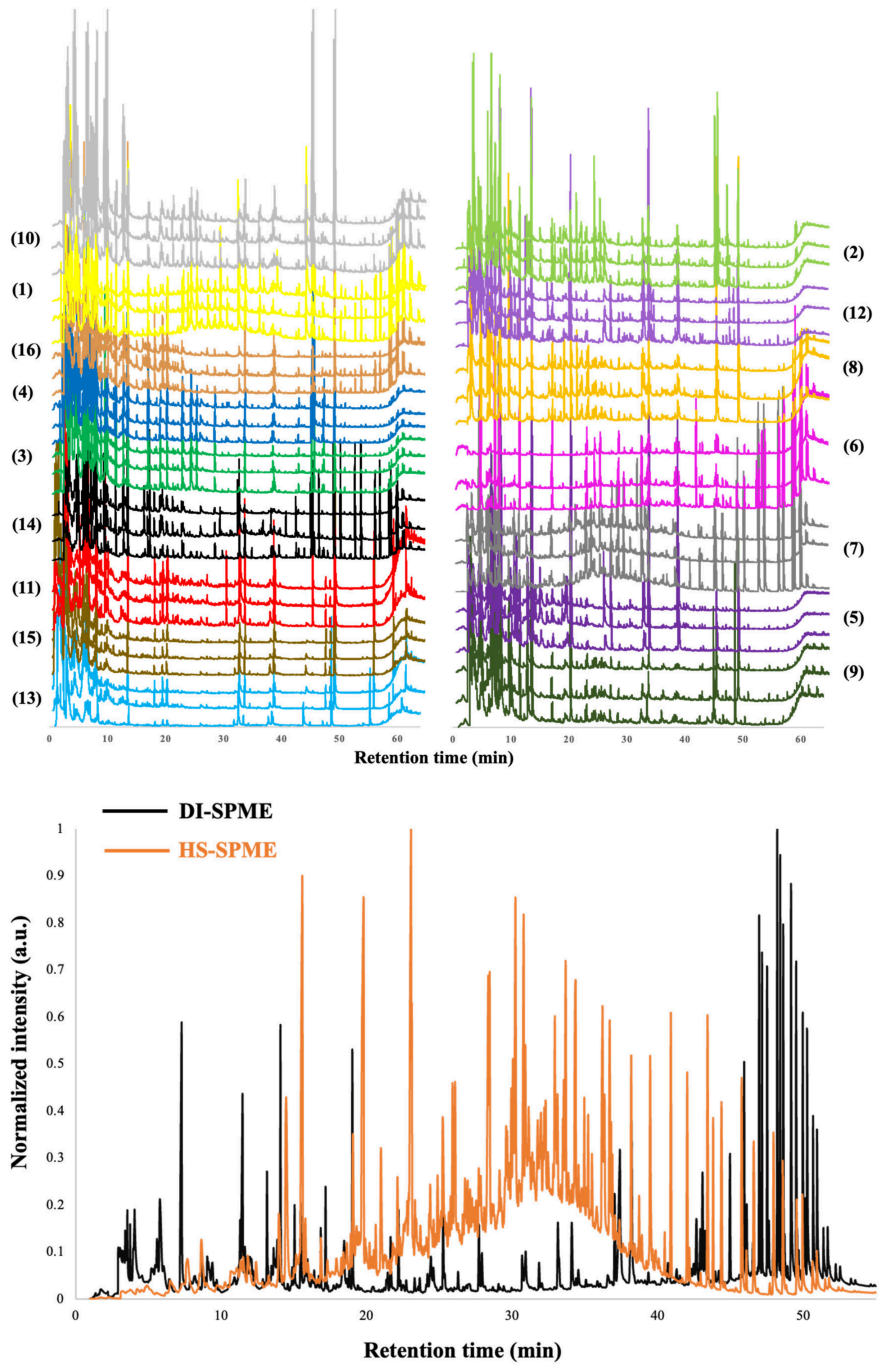
**Top**: Mezcal GC data matrix submitted to different (un)-supervised multivariate statistical analyses for non-targeted and targeted metabolomics, obtained from Method 1 (Section 2): 1–2, 5—*A. angustifolia*, Sierra Sur, Oaxaca (Ancestral); 3–4—*A. potatorum*, Sierra Sur, Oaxaca (Ancestral); 6–7—*A. salmiana*, Centro of San Luis Potosí (Artisanal); 8—*A. marmorata* Sierra Sur, Oaxaca (Artisanal); 9–10—*A. angustifolia*, Valles Centrales, Oaxaca (Artisanal); 11, 13, 15—*A. karwinskii* Sierra Sur, Oaxaca (Artisanal); 12—*A. americana* ‘Oaxacensis’, Valles Centrales, Oaxaca (Artisanal); 14, 16—*A. potatorum*, Valles Centrales, Oaxaca (Artisanal). Chromatogram color codes are equivalent for the same samples used in Method 2 (Figure 3). **Bottom**: Comparative direct immersion (black, present methodology) and standard [13] head space (orange) solid-phase microextraction gas chromatography, Method 1, applied to sample ID No. 3, Table 1.

**Figure 3:**
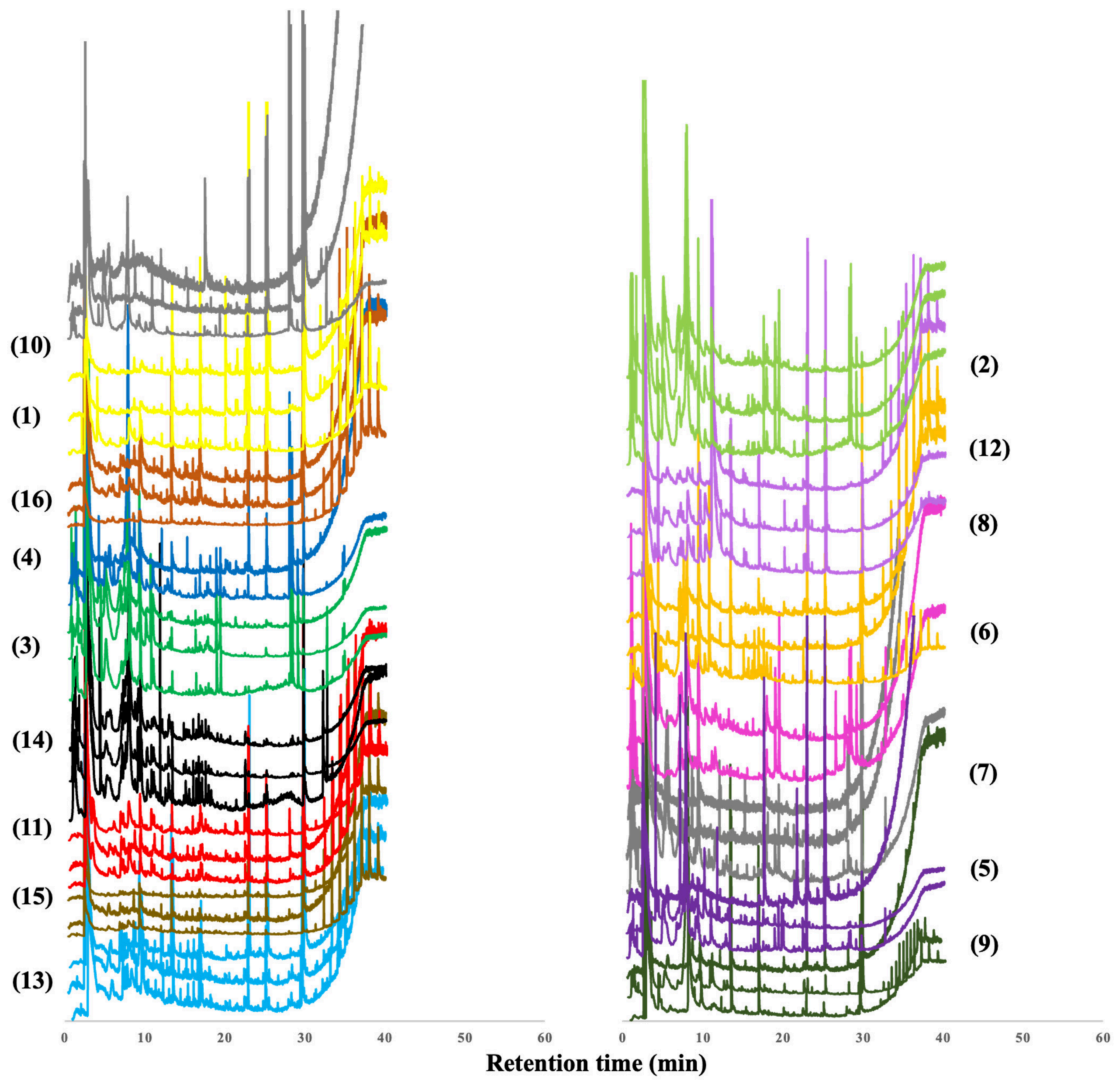
Mezcal GC data matrix submitted to different (un)-supervised multivariate statistical analysis for non-targeted and targeted metabolomics obtained from Method No. 2 (Section 2): 1–2, 5—*Agave angustifolia*, Sierra Sur, Oaxaca (Ancestral); 3–4—*Agave potatorum*, Sierra Sur, Oaxaca (Ancestral); 6–7—*Agave salmiana*, Center of San Luis Potosí (Artisanal); 8—*Agave marmorata* Sierra Sur, Oaxaca (Artisanal); 9–10—*Agave angustifolia*, Valles Centrales, Oaxaca (Artisanal); 11, 13, 15—*Agave karwinskii* Sierra Sur, Oaxaca (Artisanal); 12—*Agave americana* var. oaxacensis, Valles Centrales, Oaxaca (Artisanal); 14, 16—*Agave potatorum*, Valles Centrales, Oaxaca. Chromatogram color codes are equivalent for the same samples run with Method 1 (Figure 2).

Each GC-MS essay was performed in triplicate. Considering the number of samples from a particular geographical origin, and from each manufacturing process, using each agave species, and accounting for GC-MS repetitions, the GC-MS data matrix was constructed. We used 16 different samples (Table 1), analyzed in triplicate with GC-MS, resulting in a total of 48 chromatograms (Figure 2 and Figure 3).

### 2.2. Analytical Methods

#### 2.2.1. Alcohol Content

Alcohol content (%ABV: alcohol by volume) was measured according to the local norm, NMX-V-013-NORMEX-2019, with a set of certified hydrometers, OIML ISO 4801-NF B 35-515 (Alla France, Chemillé, France) [25]. A total of 16 mezcal samples of ancestral (5 samples, all from Sierra Sur Oaxaca) and artisanal (11 samples from all regions studied) manufacturing processes comprise the full dataset. The geographical origin dataset comprises nine samples from the Sierra Sur region, five from the Valles Centrales region (both regions in Oaxaca) and two from San Luis Potosí (center of the state). Finally, *A. angustifolia* (5), *A. potatorum* (4), *A. salmiana* (2), *A. marmorata* (1), *A. karwinskii* (3), and *A. americana* “Oaxacensis” (1) comprise the species dataset. The measured %ABV of the full set of analyzed samples ranged between 38.9 and 48.2% ABV (Table 1 and Table 2).

#### 2.2.2. Operative Settings for Volatile Compound Extractions

Volatile compounds were extracted by means of direct immersion solid-phase microextraction (DI-SPME) with a divinylbenzene, wide-carbon-range polydimethylsiloxane DVB/CWR/PDMS) fiber measuring 10 mm/80 μm in thickness (Agilent, Santa Clara, CA, USA). DI-SPME is a sensitive method that is used for the analysis of clean samples [26] such as the mezcal samples. The extraction process is summarized as follows: 1 mL of a mezcal sample, previously kept at 4 °C, was placed into a 2.0 mL amber glass vial with silicone septa cap. For Method 1 (vide infra), the sample was stirred at 1200 rpm (Vortex, Scientifics Industries Inc., Bohemia, NY, USA) for 5 min at room temperature (25 °C) prior to fiber exposition, since more volatile compounds can be extracted in such conditions [27]. This homogenization step was not included in Method 2 (vide infra). The fiber was conditioned according to the manufacturer’s instructions and was set in the manual injection holder (Supelco, Bellefonte, PA, USA). The holder was inserted through the septa and the fiber was immersed directly into the sample for one minute. This duration was sufficient to isolate a set of metabolites that produce high-resolution chromatograms with flat baselines, as needed to fingerprint the discriminant factors of the agave species, the geographical origin and the manufacturing processes. This methodology enabled the identification of 12 discriminant metabolites (vide infra); of these, 7 were esters, and we found that 71% of the retained esters are sensorially active. This characteristic is known to be important in the sensory contribution of alcoholic beverages [28], and they have been reported to be important compounds that provide key sensory notes in spirit drinks [13,29]. After extraction, the fiber was withdrawn from the holder, removed from the vial, and inserted into the GC-MS splitless injector, which was programmed with a purge flow of 50 mL/min for two minutes in Method 1 and one minute in Method 2 (vide infra). The purpose of this was to ensure the complete desorption of the analytes from the fiber and to eliminate memory effects [26]. The head space solid-phase microextraction (HS-SPME) procedure was carried out with one representative sample (sample ID No. 3, see Table 1), using the same temperature ramp (vide infra) and mobile and stationary phases as used in Method 1; however, the volatile extraction method was used here for comparative purposes. The HS-SPME-selected fiber had a 65 μm coating and was DVB-sorbent.

#### 2.2.3. Chromatographic Conditions

- GC-MS Parameters

Gas chromatography–mass spectrometry (GC-MS) was performed with a Trace 1310™ gas chromatograph coupled to a ISQ^LT^ single quadrupole mass detector (both from Thermo Scientific, Waltham, MA, USA). A J&W HP-5 ms fused silica capillary column was used, which had a 30 m × 0.25 mm internal diameter and 0.25 μm film thickness, with 5% Phenyl-methylpolysiloxane (Agilent, Santa Clara, CA, USA). The carrier gas was helium, fed at a flow rate of 1 mL min^−1^. Two separation methods were designed (referred to here as Method 1 (Figure 2) and Method 2 (Figure 3)).

- Instrumentation

**Figure 4:**
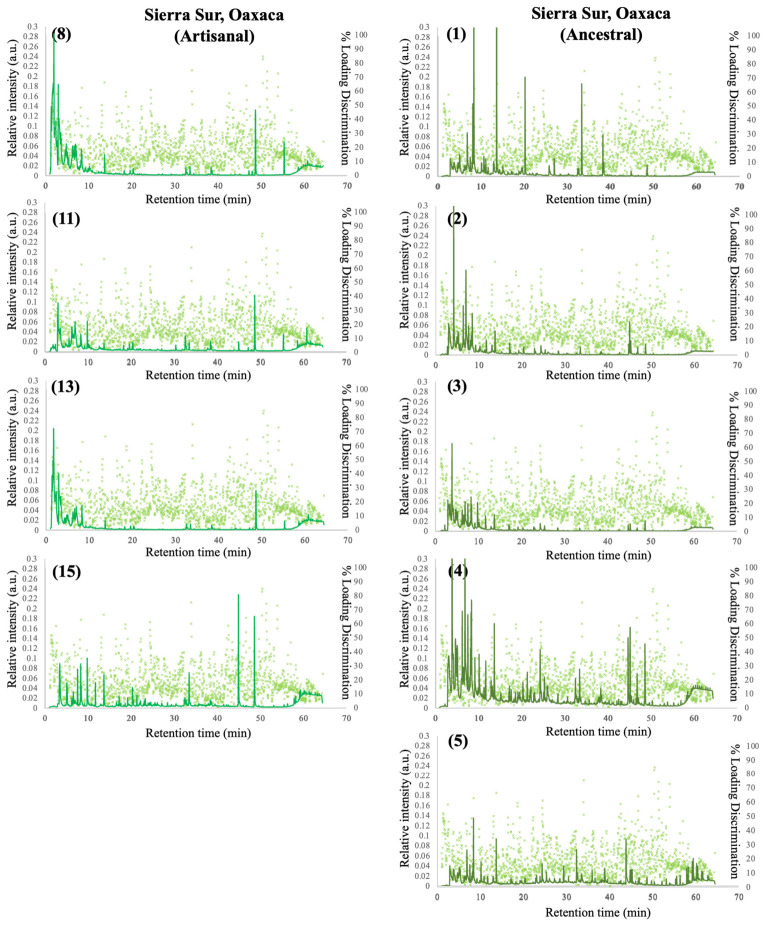

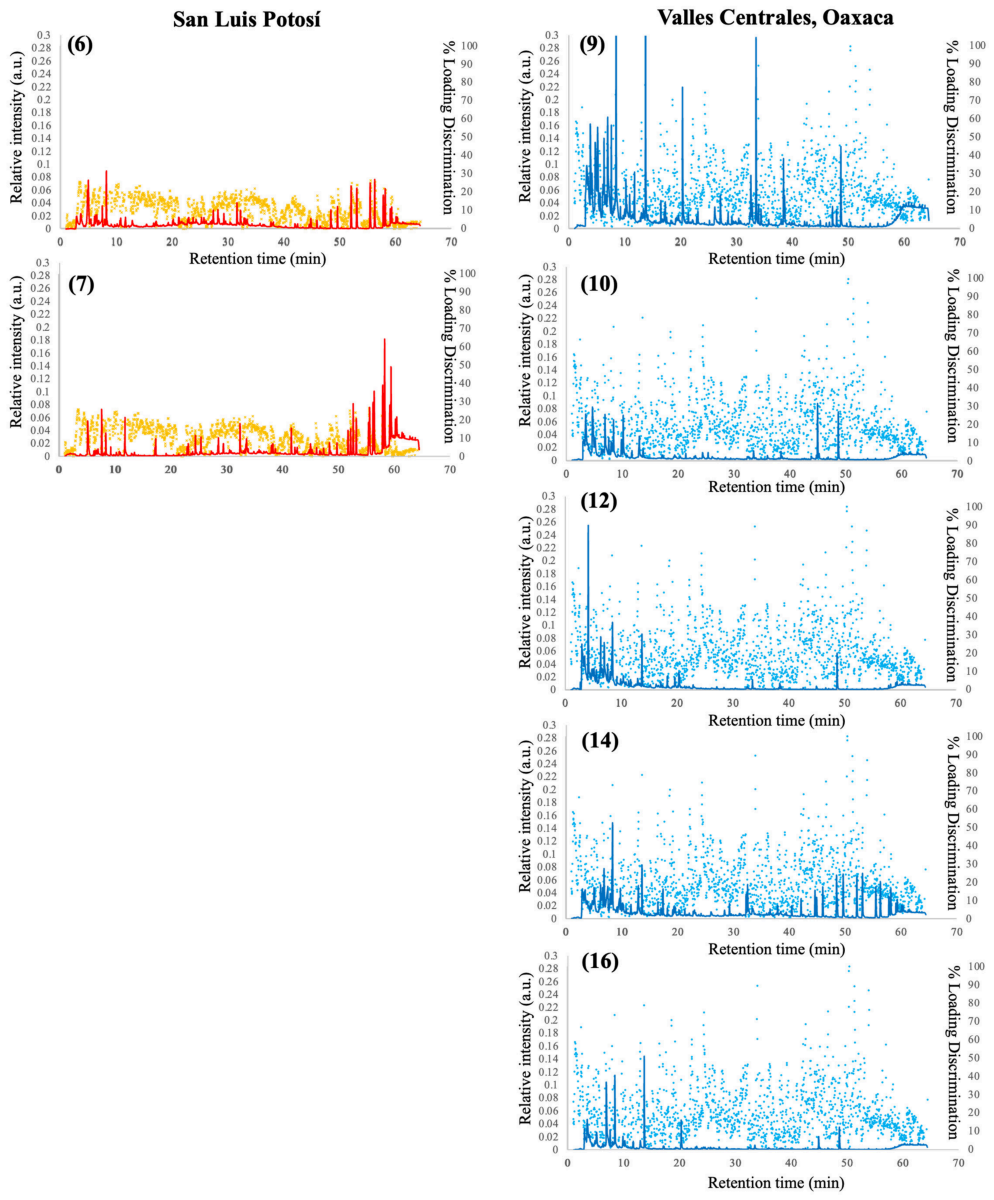

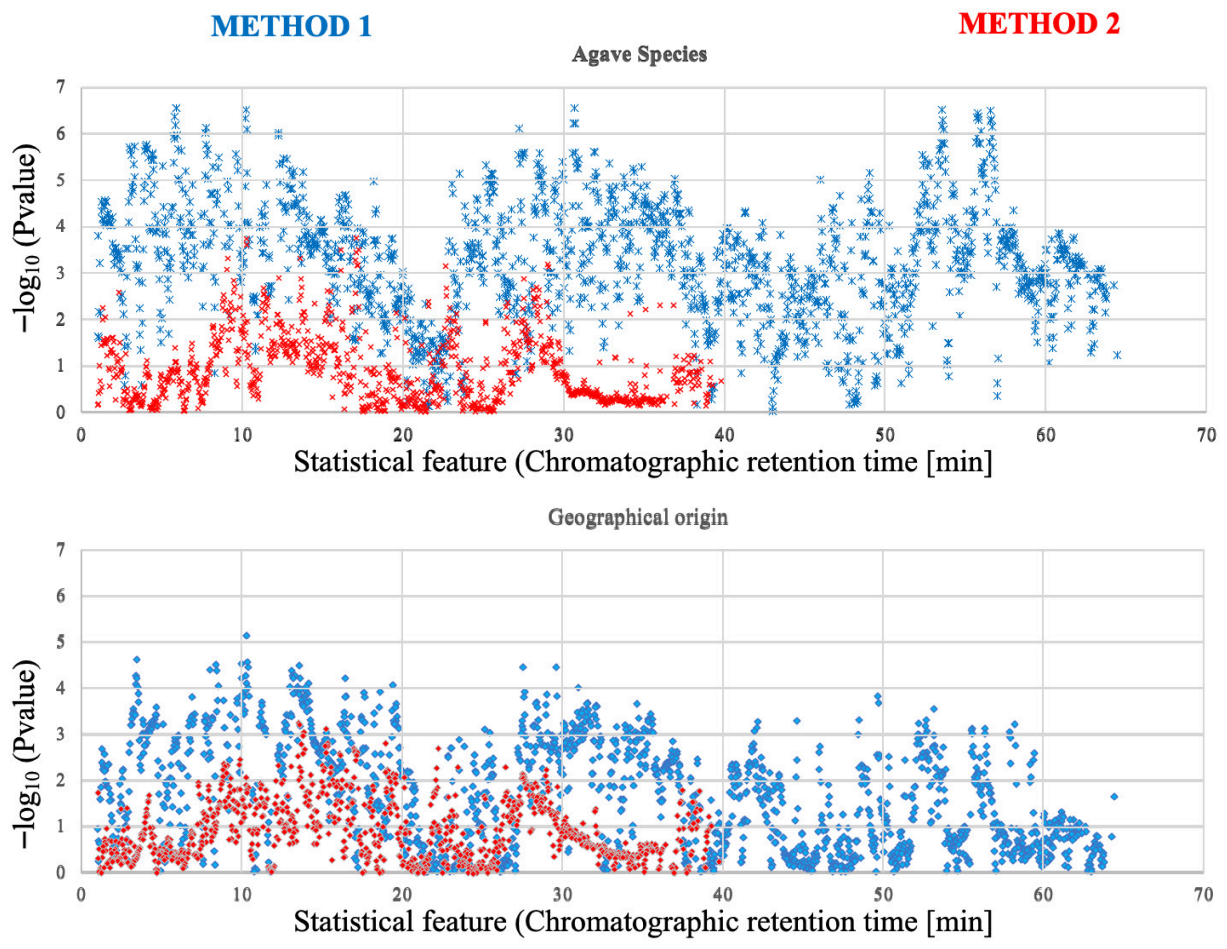
**Top** and **Middle**: Full set of GC chromatograms from 16 analyzed samples with Method 1 (see Table 1 and Figure 2. Numbering of 16 chromatograms are in agreement with series 1–16 listed in Table 1 and schemed in Figure 2 and Figure 3). Color code of experimental GC profiles are in agreement with the mezcals’ geographical origins, shown in Figure 1 and Figure 4 (vide infra): San Luis Potosí (red), Valles Centrales, Oaxaca (blue) and Sierra Sur, Oaxaca (Green). GC-normalized chromatograms are overlayed with their corresponding PLS-DA regression vector coefficients (yellow crosses for San Luis Potosí chromatograms, light blue dots for Valles Centrales chromatograms, and green crosses for Sierra sur artisanal and ancestral mezcals’ chromatograms), indicating in each case the % loading discrimination that each retention time presents for disentangling discriminant factors. **Bottom**: univariate one-way analysis of variance gas chromatography statistical features of Method 1 (blue, a total of 1693 average relevant features) and Method 2 (red, a total of 1035 average relevant features), represented as −log_10_ (*p* value) as a function of GC retention times, whereas all −log_10_ (*p* value) > 2 are statistically significant for disentangling discriminant factors.

The GC was equipped with a split–splitless injector held at 270 °C. In Method 1 (see Figure 2 and Figure 4), the oven was programed with a 4-temperature ramp program:

- Starting at 50 °C, which was held for 1 min and then increments were made up to 100 °C with a 10 °C min^−1^ rate from 2 min to 6 min.
- From 100 to 250 °C, a rate of 3 °C min^−1^ was used from 7 min to 56 min.
- From 250 to 300 °C, a rate of 15 °C min^−1^ was used from 57 min to 59 min and 20 s.
- Finally, it was held at 300 °C for 5 min, for a total chromatography time of 63 min and 25 s.

For Method 2 (see Figure 3), the oven program was run as follows: initially, it was held at 50 °C for 1 min; then, the temperature was raised from 50 to 300 °C at a rate of 7 °C min^−1^; finally, it was held at 300 °C until the end of the temperature control program (39 min and 45 s). Electronic Impact Mass Spectrometry (EI-MS) was set in TIC/Scan (Total Ion Current) mode from 50 to 500 *m*/*z* with 0.2 scan s^−1^ (Dwell) of scanning rate for Method 1 and from 50–650 *m*/*z* with 0.2 scan s^−1^ (Dwell) of scanning rate for Method 2. The transfer line and ion source temperatures were held at 270 and 200 °C, respectively. The raw data were processed using Xcalibur™ software version 4.0 (Thermo Scientific, USA).

#### 2.2.4. Data Analysis

The alcohol content data were evaluated with a one-way analysis of variance (ANOVA) for 16 mezcals according to the type of process (artisanal and ancestral), another ANOVA for 16 mezcals as a function of their geographical origin (Sierra Sur and Valles Centrales, Oaxaca, and San Luis Potosí) and finally a third ANOVA of the full set of mezcals, as a function of their agaves’ species (*A. potatorum*, *A. karwinskii*, *A. angustifolia*, and *A. salmiana*). The software SAS version 9.4 (SAS Institute, Inc., Cary, NC, USA) was used, and the differences were considered statistically significant at *p* < 0.05.

**Figure 5:**
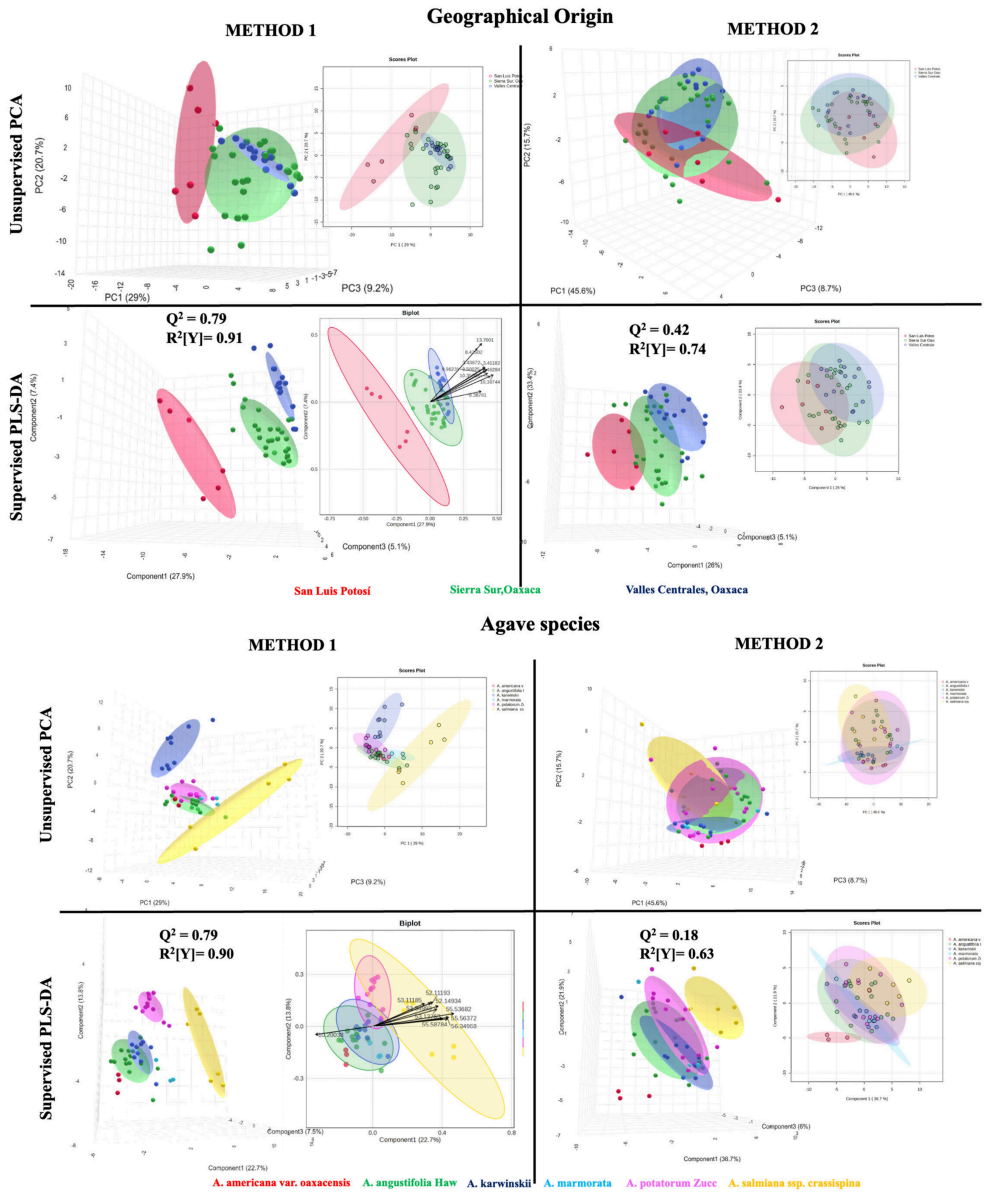

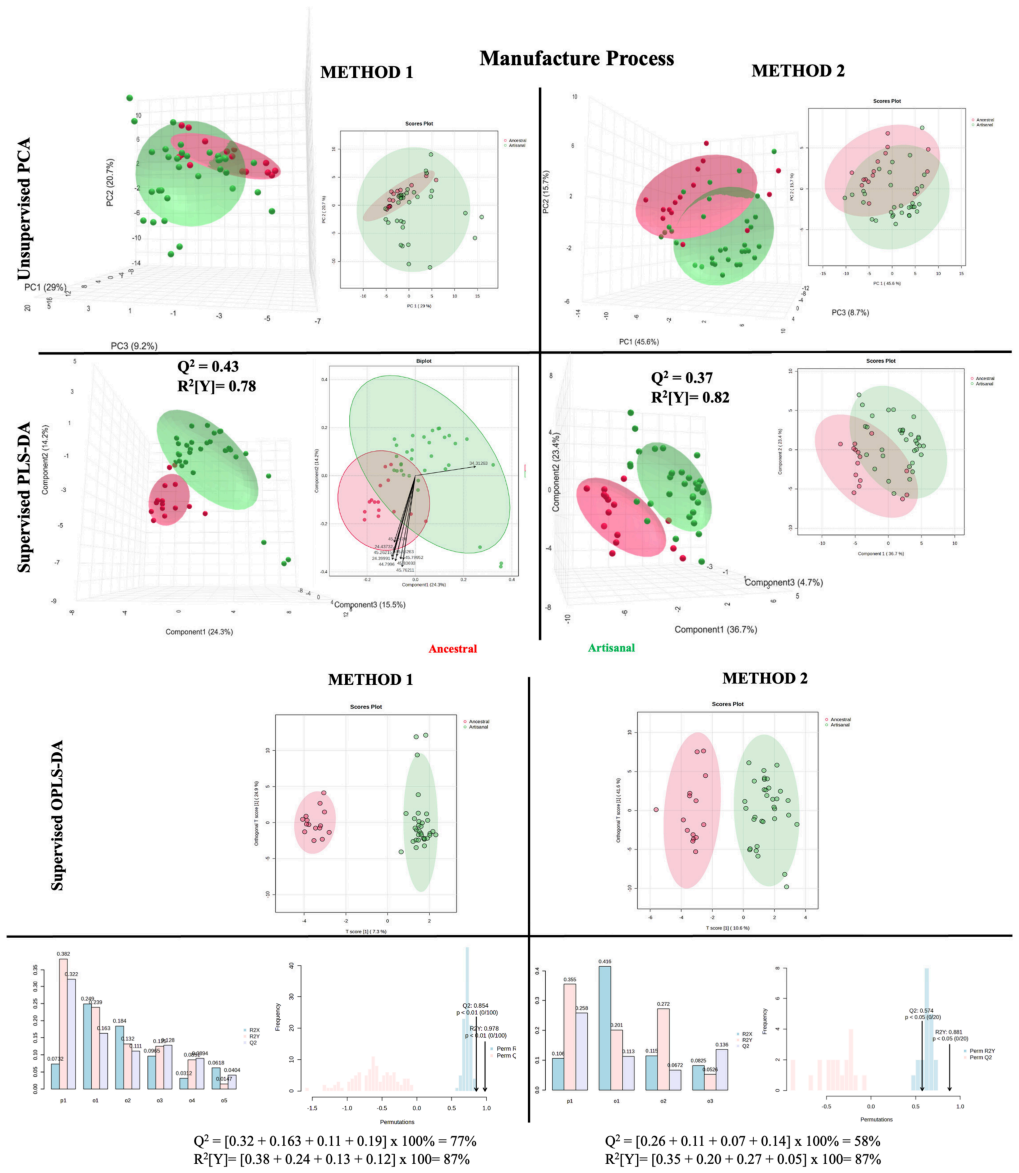
Comparative score plots obtained as MSA data outputs of Method 1 (left) and Method 2 (right) GC matrixes. **Top**—geographical origins (San Luis Potosí in red, Sierra Sur, Oaxaca in green and Valles Centrales, Oaxaca in blue); **middle**—agave species (*A. angustifolia* in green, *A. potatorum* in magenta, *A. salmiana* in yellow, *A. marmorata* in turquoise *A. karwinskii* in dark blue and *A. americana* ‘Oaxacensis’ in red); **bottom**—manufacturing processes (ancestral in red; artisanal in green). All cases include unsupervised PCA and supervised PLS-DA. For manufacturing processes, we include a pairwise OPLS-DA analysis. T2 Hotelling’s ellipses have a 95% confidence level in all cases. For PCA and PLS-DA holistic fingerprints, the explained variances are highlighted in parentheses along the axis; for the PLS-DA models, performances by means of R2 and Q2 classifications are quoted within the score plot. For OPLS-DA, the permutation analysis between one predictive (p1) and three orthogonal (o1, o2, o3) components produced the observed and cross-validated R2X (blue histograms), R2Y (pink histograms), and Q2 (violet histograms) performance coefficients. In all cases, PCA and PLS-DA score plots are represented in its two- and three-dimensional, three-principal components. Black arrows in 2D PLS-DA biplots contain both score plots and the most relevant variable importance in projection (VIP) loading vectors, showing the 15 top discriminant retention times.

The chemometrics multivariate statistical analyses of GC-MS data matrixes were performed using unsupervised principal component analyses (PCA, see Figure 5), as well as supervised partial least square discriminant analyses (PLS-DA, see Figure 5) and pairwise orthogonal projections to latent structures discriminant analysis (OPLS-DA). These approaches were used as supervised MSA confirmatory methods for discriminating between ancestral and artisanal manufacturing processing methods (see Figure 5). They were implemented with the software Metaboanalyst 6.0 [30]. The data input matrixes were composed with .zip files containing 48 chromatograms (16 mezcal samples × 3 replicates per sample) for both the Method 1 and Method 2 GC chromatogram series. Method 1 GC data matrixes (Figure 2 and Figure 4) were constructed from a basis of 895,680 total peaks (see Figure 4, bottom, blue features: the span of each GC chromatogram produced from the 1 to 64.45 min temperature ramp of Method 1, that generates 18,660 data points per sample × 48 GC chromatograms). Method 2 GC data matrixes (Figure 3) are considerably smaller. This is because each GC chromatogram was constructed from ramps that produce chromatograms with peaks between 1 and 39.8 min, for a total of 11,410 data points. When this is considered in application to the 48 chromatograms (16 samples × 3 replicates), we obtain a data matrix comprising 547,680 total peaks for Method 2 GC data inputs (see Figure 4, bottom, red features). The data pre-processing included, in all cases, non-parametric, relative standard deviation with low-variance data filters. Quantile normalization was applied for adjusting the systematic differences amongst the chromatograms within each data matrix. Each is composed of more than 1000 features per matrix. The variance-stabilizing normalization method is a mathematical data-adaptative transformation scheme for suppressing incoherent values and range-scaling factors for feature adjustment based on variable dispersion. These approaches are all applied to remove any possible variations during the experimental phase, ensuring that the features are as comparable as possible [31,32,33,34]. The multivariate statistical analyses of the GC patterns with more than two discriminant factors (geographical origin [San Luis Potosí/Valles Centrales/Sierra Sur] and agave species [*A. angustifolia*/*A. potatorum*/*A. salmiana*/*A. marmorata*/*A. karwinskii*]) were first submitted to an univariate one-way analysis of variance (ANOVA, see Figure 4, bottom) in order to classify statistical significance as features with −log_10_ (*p* value) > 2 (*p* value < 0.01). The multivariate ANOVA for the dataset, involving two discriminant factors (manufacturing processes: ancestral vs. artisanal mezcals), was computed using the OPLS-DA computational permutation procedure (Figure 5). Both PLS-DA- and OPLS-DA-supervised validations were performed with 100 permutations per analysis. The reliability of each classification per model was evaluated in terms of goodness-of-fit (R^2^) and goodness-of-prediction (Q^2^) measures. The T2 Hotelling’s regions were depicted by ellipses in score plots of each model, defining a 95% confidence interval [17,35,36].

## 3. Results

The one-way ANOVA for the processes analysis shows that the %ABV results are not significantly different (*p* > 0.05) between ancestral and artisanal mezcals. Furthermore, more important dispersions are shown in the artisanal samples (σ = ±3.2); for example, mezcals from San Luis Potosí, with lower %ABV (all xs) results, were manufactured with artisanal practices [5]. When the %ABV values of mezcals are expressed in terms of their geographical origin or by agave species, it is clear that samples from San Luis Potosí (*A. salmiana* species and all produced by artisanal practices) present a lower alcohol content (*p* < 0.05) in comparison with their Oaxaca counterparts, with %ABV results above 45%. Finally, in terms of different species and manufacturing processes from the same geographical origins (Oaxaca), the most important %ABV dispersions are observed for mezcals coming from Valles Centrales County (σ = ±2.6), indicating non-negligible variability in alcohol contents between *A. angustifolia*, *A. americana* ‘Oaxacensis’ and *A. potatorum* mezcals belonging to the region of Oaxaca.

**Figure 6:**
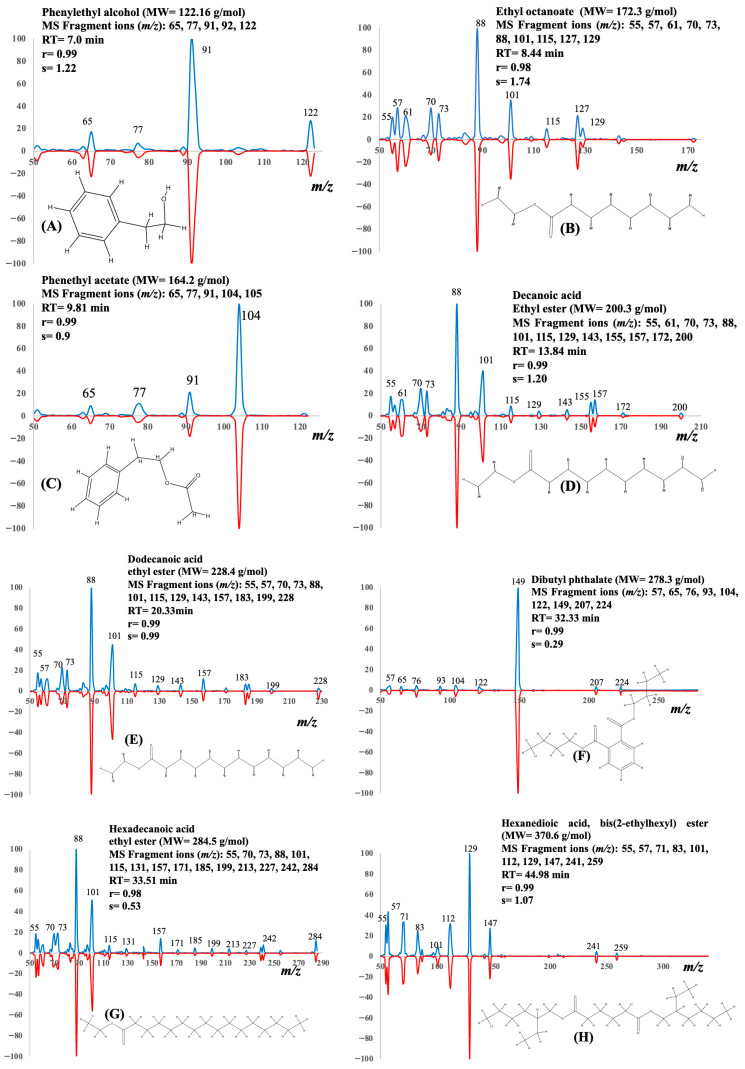

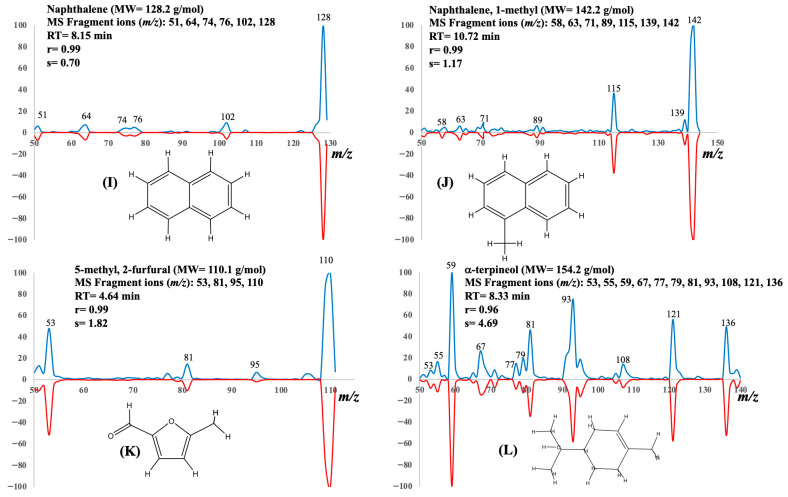
EI-MS spectra, comprising the experimental *m*/*z* fragmentation pattern (blue plots) of each of the identified discriminant retention times (in minutes): 7.0—phenethyl alcohol ((**A**) within the figure); 8.44—octanoic acid, ethyl ester (ethyl octanoate, (**B**) within the figure); 9.81—phenethyl acetate ((**C**) within the figure); 13.84—decanoic acid, ethyl ester ((**D**) within the figure); 20.33—dodecanoic acid, ethyl ester ((**E**) within the figure); 32.33—dibutyl phthalate ((**F**) within the figure); 33.51—hexadecanoic acid, ethyl ester ((**G**) within the figure); 44.98—hexanedioic acid, bis (2-ethylhexyl) ester ((**H**) within the figure); 8.15—naphthalene ((**I**) within the figure); 10.72—naphthalene, 1-methyl- ((**J**) within the figure); 4.64—5-methyl-2-furfural ((**K**) within the figure); 8.33 min—α-terpineol ((**L**) within the figure). These are shown with respect to the *m*/*z* fragmentation pattern obtained from the National Institute of Standards and Technology (NIST) Mass Spectrometry Data Center (red plots). EI-MS specular reflection continuous plots of the full set of identified metabolites present a Pearson correlation coefficient (r) between both arrays and their respective standard deviations of the difference between each relative intensity at a particular *m*/*z* range. Pearson’s correlation coefficients presented values from 0.96 to 0.99, whilst standard deviations range from 0.29 to 4.69.

Figure 2 presents full series of mezcals’ GC/DI-SPME data matrixes obtained with high-resolution Method 1, in comparison to GC/DI-SPME chromatograms with lower resolution and selectivity, depicted in Figure 3. A pairwise comparison between developing gas chromatographic separations of mezcals’ volatile compounds by their extraction with even direct immersion (DI, black in Figure 2, bottom) or head space (HS, orange in Figure 2, bottom) solid-phase microextraction (SPME) is also demonstrated. The discriminant capacity of GC/DI-SPME in the Method 1 samples was depicted by overlaying their PLS-DA regression vector coefficients over their geographical-origin-normalized gas chromatograms; this is observable for distinguishing the loading discrimination percentage of each retention time. Furthermore, the statistical significance of each retention time for disentangling discriminant factors was represented by plotting the −log_10_ (*p* value) as a function of GC retention times; in contrast, retention times with −log_10_ (*p* values) bigger than 2 (*p* absolute value < 0.01) represent statistically significant retention times. Figure 5 highlights the unsupervised PCA and supervised PLS-DA/OPLS-DA (pairwise comparison between ancestral and artisanal manufacturing processes) score plots, obtained from MSA analyses of GC/DI-SPME Methods 1 and 2. These show the supervised goodness-of-fit (R^2^) and goodness-of-prediction (Q^2^) for discriminating between the mezcals’ geographical origins, agave species and manufacturing processes. Finally, Figure 6 describes the EI-MS assignment strategy of all volatile compounds by means of EI-MS specular reflection between the *m*/*z* experimental fragmentation pattern, with respect to its National Institute of Standards and Technology (NIST) Mass Spectrometry Data Center *m*/*z* fragmentation pattern counterpart. The data show each of the twelve assigned metabolites (see Table 3) with a series of MS-fragmented ions (*m*/*z* patterns) at a particular retention time (RT, minutes). We report the Pearson correlation coefficients (r) and their respective standard deviations between both the experimental and the NIST data basis *m*/*z* patterns.

## 4. Discussion

### 4.1. Alcoholic Content

Table 2 shows that all samples are in accordance with the local requirements, NOM-070-SCFI-2016; this regulation is used to trace the specifications of alcoholic beverages, with %ABV ranges between 35 and 55% required for mezcals [64]. The importance of controlling %ABV in mezcals relies on the known fact that alcohol content plays a central role in sensorial perceptions by promoting multiple kinesthetic flavors and sensations [65,66].

### 4.2. Gas Chromatography Separation Method

The gas chromatograms presented in Figure 3, obtained by Method 2 (vide supra, see Section 2), were obtained by applying a DI-SPME in order to extract volatile compounds that can be used to obtain GC non-targeted chemometrics profiles to discriminate between manufacturing processes, geographical origins, and agave species. This approach was adapted from a previous work, extracting volatile pesticides in mangoes [27]; it was also inspired by a local Mexican norm, 340–341, for identifying and quantifying higher alcohols and organic acids in spirits [64]. A linear temperature ramp of 7 °C/min produces an experimental separation time of 39 min and 45 s. The gas chromatograms produced with Method 2 are poorly exploitable for targeted and non-targeted, unsupervised and supervised chemometrics analyses. Most of the chromatograms present important baseline distortions after 30 min of separation; however, this is a chromatographic condition that hampers the identification and selection of metabolites with the longest retention times. In turn, these show the highest affinity to the 5-phenyl-methylpolysiloxane stationary phase. In contrast, the use of a different separation method that roughly doubles the separation method time, with respect the previous method (64 min and 25 s for Method 1, with respect the 39 min and 45 s of chromatographic time for Method 2), produces exploitable data input matrixes (Figure 4) for (un)supervised multivariate statistical analysis. The increment of chromatographic resolution, selectivity and retention times amongst samples needed to obtain exploitable GC data matrixes was possible with a 4-temperature ramp program (see Section 2)

It is notable that there is an outstanding improvement in all the above-mentioned chromatographic parameters, including the baselines that allowed a peak bucketing of the discriminant peaks that elute during the first 15 min of mezcals using chromatographic essays (see Figure 2 and Figure 4). These represent 66% of the targeted metabolites needed to discriminate between ancestral and artisanal manufacturing processes, geographical origins and agave species (vide infra).

For comparison purposes, sample ID No. 3 (*A. potatorum* species, Sierra Sur Oaxaca geographical origin, ancestral manufacturing process, see Table 1) was analyzed with both Method 1 DI-SPME GC and an HS-SPME GC-equivalent method. As expected, the HS-SPME approach is unambiguously better in extracting a set of metabolites mostly in the retention time range of 20–40 min (see Figure 2, bottom). Because of this, the HS-SPME gas chromatograms present baseline distortions that could obstruct MSA treatments in validating the fingerprints of agave species, geographical origins and manufacturing processes. As a result, a DI-SPME GC scheme was selected; here, we sacrificed the advantages gained in targeted chemometrics offered by HS-SPME in favor of having better baselines; in turn, we achieved chromatographic resolutions that maximize the quality of the untargeted GC Method 1 fingerprints.

Each method in turn produces 48 GC chromatograms (16 samples × 3 replicates), represented as a certain number of data points that define each chromatogram. For Method 1, the average number of data points is 18,660; for Method 2, the average number of data points is 11,410; these represent chromatograms of 64 min 25 s and 39 min with 45 s, respectively, each with a span of 0.0034 min per data point. In turn, the combined number of data points (18,660 Method 1/11,410 Method 2), the number of mezcal samples (16) and number of replicates per sample involve MSA data matrixes of 895,680 and 547,680 data points, respectively. The GC Method 1 and Method 2 cohorts were then stored in three separate .zip folders (Method1-Mezcal-GCMS-GeoOrigin.zip, Method1-Mezcal-GCMS-Processes.zip, Method1-Mezcal-GCMS-Species.zip, Method2-Mezcal-GCMS-GeoOrigin.zip, Method2-Mezcal-GCMS-Processes.zip, and Method2-Mezcal-GCMS-Species.zip; see data availability statement). This supported the one-factor statistical analysis. After we implemented a low-variance data filter and GC feature normalization, the machine learning inputs described more than two variables within the discriminant factor: GeoOrigin [San Luis Potosí/Valles Centrales Oaxaca/Sierra Sur Oaxaca] and Species [*Agave potatorum*/*Agave angustifolia*/*Agave karwinskii*/*Agave salmiana*/*Agave marmorata*/*Agave americana* ‘Oaxacensis’]. These were submitted to an univariate one-way analysis of variance (ANOVA, see Figure 4, bottom). This was used to analyze the statistical significance of each GC data point. Roughly 9% of the total number of GC data points per matrix were described as the most discriminant features (1693/18,660 statistically significant data points for Method 1 and 1035/11,410 statistically significant data points for Method 2). In turn, these present a −log_10_ (*p* value) bigger than 2 (*p* < 0.01). Interestingly, supervised PLS-DA average loading plots, overlayed with GC chromatograms obtained with Method 1, match their % loading discrimination with ANOVA −log_10_ (*p* value) > 2 relevant statistical features. The statistical significance of the GC features contained in the data matrixes describing the two manufacturing processes (ancestral and artisanal) were calculated as *p* < 0.01 statistical significance (Method 1) and *p* < 0.05 statistical significance (Method 2). These were used for the pairwise OPLS-DA permutation analysis between one predictive (p1) and four orthogonal (o1, o2, o3, o4) components. In turn, these were produced between the observed and cross-validated validated goodness-of-fit [R2X, R2Y] and goodness-of-prediction [Q2] performance coefficients (vide infra, see Figure 5, bottom: OPLS-DA permutation tests).

### 4.3. GC Non-Targeted Chemometrics

Figure 5 depicts a series of unsupervised PCA as well as supervised OPLS-DA and PLS-DA machine learning multivariate statistical analysis score plots of mezcals’ GC data matrix obtained with separation Method 1 (left in Figure 5) and Method 2 (right in Figure 5). In all cases, 2D- and 3D-PCA were used as exploratory tools for evaluating the discriminant capacity of each of the six analyzed GC data input cohorts (see Section 2 and Data Availability Statement)

First, the orthogonal projections for the latent structures discriminant analysis (OPLS-DA) with the use of one predictive (p1) and four orthogonal (o1, o2, o3 and o4) components (Figure 5, left bottom) was used for the pairwise discriminations between the artisanal and ancestral manufacturing processes of the full set of analyzed mezcals (Table 1); these obtained scores for the goodness-of-fit and the goodness-of-global prediction of R^2^[Y] = 0.87 and Q^2^ = 0.77, respectively. These scores were obtained when mezcals where analyzed with GC Method 1. The scores were significantly lowered by Q^2^ = 0.58 and R^2^[Y] = 0.87, respectively, with GC Method 2 data inputs (Figure 5, right bottom). The artisanal process for manufacturing the San Luis Potosí mezcals is comparable to several processes reported in Oaxaca based on the use of earthen pit ovens; these are fueled with wood and lined rocks for the agave cooking stage [37,38]. As observed in Figure 5, bottom, three principal component PLS-DA tests also differentiated between the artisanal and ancestral mezcals with GC Method 1 (Q^2^ = 0.43/R^2^ = 0.78) and, to a lesser degree, with GC Method 2 (Q^2^ = 0.37/R^2^ = 0.82). However, the imbalance between the ancestral (5 samples from the cohort, all of them from Sierra sur, Oaxaca) and artisanal (11 samples from all analyzed geographical origins and agave species) samples from the present cohort means that we must conduct a broader and more balanced ancestral sampling using Method 1 in future studies; this will allow us to discriminate between ancestral and artisanal mezcals.

The supervised partial least square discriminant analysis (PLS-DA) model was used to differentiate between the mezcals’ geographical origins, as depicted in Figure 5 (top). The discrimination accuracy for the mezcals from Oaxaca and San Luis Potosí (22°9′4″ N, 100°58′34″ W), as well as for distinguishing between Oaxaca counties Valles Centrales (17°4′59.88″ N, 96°45′0″ W) and Sierra Sur (19°42′0″ N, 96°52′0″ W), was R^2^ = 0.79 and Q^2^ = 0.91 with PLS-DA approach when GC data were analyzed with Method 1. Meanwhile, the discriminant capacity of the data produced with GC Method 2 is significantly lowered to R^2^= 0.74 and Q^2^ = 0.42. As observed, it is straightforward to distinguish between mezcal samples from Oaxaca and San Luis Potosí, as there is a considerable distance between these two regions (around 1000 km) (Figure 1). However, the diversity in mezcals found in Oaxaca (as the most important mezcal producer globally [5,12,65]) requires the development of a robust machine learning model; this would allow us to discriminate between ancestral and artisanal mezcals produced in counties from the same region: Valles Centrales Oaxaca (blue scores in Figure 5; delimiting samples 9, 10, 12, 14 and 16 (Table 1) from PLS-DA score plots), Sierra Sur, and Oaxaca (green scores in Figure 5, for samples 1, 2, 3, 4, 5, 8, 11, 13 and 15 (Table 1) from PLS-DA score plots). Three-dimensional PLS-DA score plots produced from Method 1 (representing 27.9% + 7.4% + 5.1% = 40.4% of the explained variance) perfectly separate the features of both types of mezcals (Valles Centrales and Sierra Sur) from the same geographical origin: the State of Oaxaca. With a significantly lower degree of separation, the 3D-PLS-DA results produced using the Method 2 GC cohorts can modestly discriminate between geographical regions. However, the geographical origins can only be disentangled with an unsupervised PCA approach by using the GC Method 1 cohort. Such a trend can be associated with the common mezcal manufacturing practices carried out in Oaxaca, the proximity between both municipalities (around 50 km), the equivalent environmental and climatological conditions, the genetic similarity amongst agave species, and (to a lesser extent) the traits associated with authenticity and/or quality [67,68]. However, the GC PLS-DA for distinguishing between the mezcals’ geographical origins, herein presented, can be the basis for obtaining a Geographical Indication (GI) test. Such tests are typically used in wine marketing for associating consumers’ consumption trends and quality with a geographical distinction [69].

Figure 5, middle, highlights the partial least square discriminant analysis of the GC data matrix to differentiate between mezcals produced with *A. angustifolia*, *A. potatorum*, *A. salmiana*, *A. marmorata*, *A. karwinskii*, and *A. americana* ‘Oaxacensis’ species. A robustness for mathematical fitting of R^2^= 0.9 and a prediction capacity of Q^2^ = 0.79 were obtained using the GC Method 1 machine learning data inputs. This is in clear contrast with its PLS-DA Method 2 counterpart, which can only predict a separation amongst the species with a score of Q^2^ = 0.18. Complementary to the last observation, only the unsupervised PCA from GC Method 1 data input matrix can easily discriminate between agave species. *A. angustifolia*’s mezcals are produced broadly as their cultivars are found from Sonora and Tamaulipas in Northern México, down to Panamá. This is in clear contrast to the *A. potatorum*, *A. marmorata* and *A. americana* ‘Oaxacensis’ varieties (Oaxaca) and *A. salmiana* (San Luis Potosí and Zacatecas) that are wild endemic species [70,71,72]. *A. karwinskii* mezcals, known as species of complex morphological complexity and diverse ethno taxa, are endemic from a broad side of Southern México; these might be considered as an intermediate case [73]. Colloquially known as “cuishe”, “madrecuishe” and “tobasiche”, *A. karwinskii* species might extend to roughly 40 different wild varieties, mostly from Puebla and Oaxaca in Southeastern México [74]. As observed with the GC Method 1 PCA and the PLS-DA, with a model robustness of 79%, unambiguous distinctions can be achieved for *A. potatorum* (from both Valles Centrales and Sierra Sur of Oaxaca) and *A. salmiana* (San Luis Potosí) species. *A. marmorata* and *A. americana* ‘Oaxacensis’ species present a statistical similarity with *A. angustifolia* that can be slightly differentiated from the others. Less evident is the discrimination between *A. angustifolia* and the analyzed *A. karwinskii* species. Interestingly, *A. angustifolia* samples from Sierra Sur, Oaxaca (samples’ ID. No. 1 and 2, Table 1), presents a clear PLS-DA proximity with *A. karwinskii* species labeled as 11, 13 and 15, all of them from Sierra Sur, Oaxaca; however, there was no proximity with the *A. angustifolia* sample, labeled as No. 5 in Table 1. This strongly suggests that sample 5 (Table 1) might be considered to be a reliable *A. angustifolia* sample from Sierra Sur, Oaxaca; No. 15 might be in concordance as a representative *A. karwinskii* mezcal from Sierra Sur, Oaxaca. In contrast, the last hypothesis undoubtedly requires further verification with DNA barcoding and/or verifiable certificates of origin. For the rest of the above-mentioned samples (1 for *A. angustifolia*, 13 for *A. karwinskii*, and more severely for sample 2 *A. angustifolia* and sample 13 for *A. karwinskii*), we might be skeptical of the labelled species given by the model.

The importance of fingerprinting mezcals’ manufacturing processes, geographical origins and agave’s species with reliable data matrices and robust supervised multivariate statistical analysis is illustrated by the comparison of the present machine learning GC model and recent mezcals’ metabolomics studies, performed with nuclear magnetic resonance spectroscopy [5]. In terms of data matrix untargeted fingerprinting capacities, it was recently demonstrated that mezcals’ non-volatile MSA data matrixes (obtained with ^1^H noesy presat NMR spectroscopy and further treated with supervised OPLS-MSA) might partially discriminate three different geographical origins (Q^2^ = 0.822); to a lesser extent, they may determine agave species (Q^2^ = 0.504) or ancestral and artisanal manufacturing processes (Q^2^ = 0.178) [5}. In contrast, the herein-presented data matrix, obtained with gas chromatography separation Method 1, can much better discriminate between the ancestral and artisanal manufacturing processes (Q^2^ = 0.77 by OPLS-DA, see Figure 5, bottom), geographical origins (Q^2^ = 0.79), and agave species (Q^2^ = 0.79). The latter discriminant factors were better described with a more modest PCA unsupervised approach, indicating the robustness and quality of the GC separation method. (Un)supervised MSA score plots constructed from GC Method 2 (right in Figure 5) produce modest–null discriminations mostly for agave species; they also derive a sort of partial discrimination for geographical origins and traditional manufacturing processes for mezcals. In contrast, the use of a non-supervised PCA approach for analyzing the shorter GC data matrix obtained with Method 2 is not satisfactory for any of the discriminant factors, evidencing the importance of the data matrix quality. Finally, the non-supervised PCA treatment for GC data matrix obtained with Method 1 shows a modest but unambiguous trend of discriminating between different geographical origins and agave species. Interestingly, the PCA score plot proposed to discriminate between mezcals’ geographical origins shows a separation between samples from San Luis Potosí and Oaxaca; in contrast, samples from the same geographical origin but different counties (Sierra Sur and Valles Centrales, Oaxaca) are discriminated between, but this is to a limited degree.

### 4.4. GC Targeted Chemometrics

The mezcals’ volatile analytes, identified by DI-SPME-GC-MS, were eluted at 4.64, 7.0, 8.15, 8.33, 8.44, 9.81, 10.72, 13.84, 20.33, 32.33, 33.51 and 44.98 min; these are detailed in Table 3. Interestingly, loading data from the targeted mezcals’ metabolites (comprising alcohols, benzene derivatives, furan derivatives, α-terpineol and roughly 70% of identified esters) were eluted after 20 min of the GC separation Method 1. In turn, these represent 66% of the targeted metabolites needed to discriminate between ancestral and artisanal manufacturing processes, geographical origins, and agave species. All the identified metabolites which eluted after 20 min of GC separation Method 1 were esters. Table 3 also presents the updated reported sensory notes of each identified alcohol, ester, benzene and furan derivative and terpene that can be produced in the agave-cooking, must-fermentations and/or liqueur-distillation stages of the manufacturing process [75,76,77,78,79]. The variable importance in projection (VIP) loading scores calculations (see the 2D PLS-DA loading biplots per discriminant factor in Figure 5) revealed that the identified markers to specific discriminant factors are as follows:

- Phenylethyl alcohol (rt = 7.0 min), octanoic acid ethyl ester (rt = 8.44 min), phenethyl acetate (rt = 9.81 min), dodecanoic acid ethyl ester (rt = 20.33 min), hexanedioic acid, bis (2-ethylhexyl) ester (rt = 44.98 min) and 5-methyl-2-furfural (rt = 4.64 min) can be used to discriminate between manufacturing processes.
- Octanoic acid ethyl ester (rt = 8.44 min), phenethyl acetate (rt = 9.81 min), decanoic acid ethyl ester (13.84 min), naphthalene (rt = 8.15 min), and 5-methyl-2-furfural (rt = 4.64 min) can be used to discriminate between geographical origins.
- Naphthalene 1-methyl (rt = 10.72 min) and α-terpineol (rt = 8.33 min) are discriminant metabolites that can be used to discriminate between agave species.

Finally, the strategy for identifying metabolites by comparison of EI-MS *m*/*z* profiles is described in Section 4.5.

### 4.5. EI-MS Targeted Analysis

From the loading plots overlayed with the GC chromatograms obtained with Method 1, expressed in Figure 4, it can be deduced that the mezcal analytes that elute at 4.64, 7.0, 8.15, 8.33, 8.44, 9.81, 10.72, 13.84, 20.33, 32.33, 33.51 and 44.98 min of separation Method 1 can be used to discriminate between either manufacturing processes and/or geographical origin and/or agave’s species. As broadly discussed within the literature [80], the identification of metabolites (mostly unknown or not reported in databases) is the most important challenge of modern metabolomics. Herein, we present the selected strategy for confirming the presence of reported metabolites that were found to be in statistical agreement with the National Institute of Standards and Technology (NIST) Mass Spectrometry Data Center [81]. This was confirmed by comparing its low-resolution Electronic Impact Mass Spectrometry (EI-MS) profiles reported at the public repository (A-L electronic impact red plots, Figure 6); we accounted for the mezcals’ experimental EI-MS *m*/*z* patterns for the f targeted metabolites, obtained at specific retention times (A-L electronic impact blue plots, Figure 6) and eluted in GC Method 1 (Section 2) at 4.64, 7.0, 8.15, 8.33, 8.44, 9.81, 10.72, 13.84, 20.33, 32.33, 33.51, and 44.98 min. Both the experimental (blue *m*/*z* patterns in Figure 6) and NIST (red *m*/*z* patterns in Figure 6) EI-MS spectra are plotted as specular *m*/*z* patterns in a continuous range. Moreover, to evaluate the consistency and the dispersion of each of the twelve EI-MS comparative fragmentation patterns (Figure 6.), we calculated the Pearson correlation coefficient between both arrays and their respective standard deviation of the difference between each relative intensity at a particular *m*/*z* range. The Pearson’s correlation coefficient (r) achieved values from 0.96 to 0.99, indicating a high match between both spectra. The standard deviations ranged from 0.29 to 4.69. In this way, twelve metabolites were identified in the mezcal samples (Table 3): phenylethyl alcohol (rt: 7.00 min, s = 1.22, r = 0.99); octanoic acid, ethyl ester (rt: 8.44 min, s = 1.74, r = 0.98); phenethyl acetate (rt: 9.81 min, s = 0.90, r = 0.99); decanoic acid, ethyl ester (rt: 13.84 min, s = 1.20, r = 0.99); dodecanoic acid, ethyl ester (rt: 20.33 min, s = 0.99, r = 0.99); dibutyl phthalate (rt: 32.33 min, s = 0.29, r = 0.99); hexadecanoic acid, ethyl ester (rt: 33.51 min, s = 0.53, r = 0.98); hexanedioic acid, bis(2-ethylhexyl) ester (rt:44.98 min, s = 1.07, r = 0.99); naphthalene (rt: 8.15 min, s = 0.70, r = 0.99); naphthalene, 1-methyl- (rt: 10.72 min, s = 1.17, r = 0.99); 5-methyl, 2-furfural (rt: 4.64 min, s = 1.82, r = 0.99); and α-terpineol (rt: 8.33 min, s = 4.69, r = 0.96).

Phenylethyl alcohol and phenethyl acetate were also identified in mezcals by experimental NMR [5]. Phenylethyl alcohol produces pleasant notes (flowers, roses, honey, and sweetness [82,83]) and phenethyl acetate is regarded as producing caramelized and floral flavors [84]. Higher alcohols, ethyl esters and acetates are formed by either yeast or bacteria metabolism during fermentation [85]. The yeast strain is one of the most important factors affecting esters’ production [74] and the sensory quality of distillates decreases when increasing esters’ number of carbon atoms [86]. The herein-reported esters, which are the major chemical type of the identified analytes, are known to contribute fruity and floral flavors [13].

Phthalates are plasticizers that can migrate from any material used in the production to produce mezcal. Although they do not cause pronounced changes in flavor, in wine, they can reduce its limpidity and freshness [87]. In general, the phthalate esters are usually clear liquids, some with faint sweet odors [83]. The dibutyl phthalate has also been reported in tequila [83], the most emblematic spirit of México.

The naphthalene was reported in agave-based alcoholic beverages such as bacanora, raicilla, sisal, tequila [72] and mezcal [12]; the naphthalene 1-methyl- was reported in sotol [84], which is produced with a similar process to the more common artisanal spirits: tequila and mezcal. On the other hand, the 5-methyl, 2-furfural (MF) is a Maillard compound produced during the cooking process of agave piñas [60]. Although the presence of furans such as MF in spirits is mainly associated with risks to human health [85], its importance is also due to its contribution to the sensory profile by imparting nutty and spicy odorant notes [84].

The terpenes have their origin in agave plants; however, throughout the mezcal production process, these compounds are transformed during the fermentation, distillation and aging steps [78]. For instance, α-terpineol has been previously identified in agave products [12].

The following components present a medium level of odor strength: octanoic acid, ethyl ester; phenethyl acetate; decanoic acid, ethyl ester; dodecanoic acid, ethyl ester; phenylethyl alcohol and 5-methyl-2-furfural [6,42,44,46,48,59]. Despite the low–medium values of odor strength for these compounds, their presence is relevant because they harmonically synergize to produce the characteristic flavor of mezcal [72].

## 5. Conclusions

This study presents a gas chromatography high-resolution separation method (Method 1) for obtaining exploitable chromatograms for GC-MS non-targeted and targeted metabolomics; these can be used as data matrixes for fingerprinting and identifying discriminant metabolites to differentiate between *A. potatorum*, *A. angustifolia*, *A. karwinskii*, *A. salmiana*, *A. marmorata* and *A. americana* ‘Oaxacensis’. These are the most commercial species of agave used in the production of mezcal. This approach can also be used to discriminate between geographical origins (San Luis Potosí and Oaxaca) and between the ancestral and artisanal manufacturing processes of mezcals. The approach proposed here must be verified with a bigger and more balanced cohort. Twelve metabolites were identified from the OPLS-DA and PLS-DA loading plots, which were confirmed with Electronic Impact Mass Spectrometry (EI-MS) mirror image mass-profile analyses. The derived Pearson’s correlation coefficients presented values in a range of 0.96 to 0.99, whilst the corresponding standard deviations were from 0.29 to 4.69. Both parameters are indicators of an acceptable correlation between experimental spectra and NIST Mass Spectrometry Data Center entries. The supervised multivariate statistical analyses loading biplots revealed discriminant metabolites for distinguishing between the manufacturing processes (phenylethyl alcohol, dodecanoic acid ethyl ester and hexanedioic acid, bis (2-ethylhexyl) ester), geographical origins (decanoic acid ethyl ester and naphthalene) and agave species (naphthalene 1-methyl and α-terpineol) used in the production of mezcals. Further studies are invited to use GC Method 1, combined with supervised MSA for discriminating between geographical origins, agave species, manufacturing processes or other relevant discriminant factors with a bigger cohort. Suh measures would avoid the possible pseudo-replication statistical issues of the current study, produced by our limited cohort (16 samples × 3 technical replicates). By extending the size of the present GC-MS chemometrics database, and due to its ease of use and the feasibility of its implementation throughout research and control laboratories, the present method might serve as a starting point for implementing control origin appellations in representative spirits throughout other regions. The proposed approach provides a food-based fingerprinting metabolomics approach for certifying specific denominations and characteristics.

## Figures and Tables

**Table 1:** Set of mezcal sampling used for DI-SPME-GC-MS analysis arranged by agave species, origin, manufacturing processes and state.

ID	Agave Species	Origin	Process	State	%ABV ^2^
1	*A. angustifolia*	Sierra Sur	Ancestral	Oaxaca	46.9
2	*A. angustifolia*	Sierra Sur	Ancestral	Oaxaca	47.1
3	*A. potatorum*	Sierra Sur	Ancestral	Oaxaca	48.2
4	*A. potatorum*	Sierra Sur	Ancestral	Oaxaca	48.2
5	*A. angustifolia*	Sierra Sur	Ancestral	Oaxaca	46.7
6	*A. salmiana*	Centro, SLP ^1^	Artisanal	SLP	39.8
7	*A. salmiana*	Centro, SLP	Artisanal	SLP	38.9
8	*A. marmorata*	Sierra Sur	Artisanal	Oaxaca	46.0
9	*A. angustifolia*	Valles Centrales	Artisanal	Oaxaca	44.4
10	*A. angustifolia*	Valles Centrales	Artisanal	Oaxaca	41.9
11	*A. karwinskii*	Sierra Sur	Artisanal	Oaxaca	45.7
12	*A. americana* ‘Oaxacensis’	Valles Centrales	Artisanal	Oaxaca	48.0 ^3^
13	*A. karwinskii*	Sierra Sur	Artisanal	Oaxaca	46.0
14	*A. potatorum*	Valles Centrales	Artisanal	Oaxaca	48.0 ^3^
15	*A. karwinskii*	Sierra Sur	Artisanal	Oaxaca	47.6
16	*A. potatorum*	Valles Centrales	Artisanal	Oaxaca	45.5

^1^ San Luis Potosí. ^2^ ABV: alcohol by volume, corrected for 20 °C. ^3^ Value according to the label.

**Table 2:** Mezcal samples’ % ABV (alcohol content) from Oaxaca and San Luis Potosí, México.

Process	%ABV
Ancestral	47.4 a ^1^ ± 0.7
Artisanal	44.7 a ± 3.2
Geographical origin	%ABV
Sierra Sur, Oaxaca	46.9 a ± 0.9
Valles Centrales, Oaxaca	45.6 a ± 2.6
San Luis Potosí	39.4 b ± 0.6
Agave species	%ABV
*A. potatorum*	47.5 a ± 1.3
*A. karwinskii*	46.4 a ± 1.0
*A. angustifolia*	45.4 a ± 2.3
*A. salmiana*	39.4 b ± 0.6

^1^ Different letters in the same column indicate significant differences according to least significant difference (LSD) test (*p* < 0.05).

**Table 3:** EI-MS identified compounds (See Figure 6) eluted at specific retention times (rt), with sensory notes, reported odor strengths, and sources of the metabolites reported in the literature. These are presented in comparison with the data obtained in the present study (*), as shown in the 2D PLS-DA biplots revealing variable importance in projection (VIP) loading vectors, showing the 15 top discriminant retention times (in Figure 5).

Compound (NIST MS No.)	rt (min)	Sensory Note	Odor Strength	Discriminant Marker
Phenylethyl alcohol (118543) [37]	7.00	Rose/honey/fresh/sweet [38]	Medium	Process/fermentation [39,40] Process (*)
Octanoic acid ethyl ester (229103) [41]	8.44	Banana/waxy [42]	Medium	Process/fermentation [39,40] Process/Geo. Origin (*)
Phenethyl acetate (228405) [43]	9.81	Floral/honey [44]	Medium	Process/fermentation [39,40] Process/Geo. Origin (*)
Decanoic acid ethyl ester (229107) [45]	13.84	Apple/waxy [46]	Medium	Process/fermentation [39,40] Geo origin (*)
Dodecanoic acid, ethyl ester (229100) [47]	20.33	Fruity/waxy/ Soapy [48]	Medium	Process/fermentation [39,40] Process (*)
Dibutyl phthalate (114974) [49]	32.33	Not reported	Not reported	Contamination [40]
Hexadecanoic acid, ethyl ester (233204) [50]	33.51	Fruity/Waxy/buttery [51]	Low	Process/fermentation [39,40]
Hexanedioic acid, bis (2-ethylhexyl) ester (291314) [52]	44.98	Not reported	Not reported	Contamination [53] Process (*)
Naphthalene (228342) [54]	8.15	Resinous/pungent [55]	Not reported	Not reported Geo. Origin (*)
Naphthalene 1-methyl (291511) [56]	10.72	Medicinal/chemical [57]	Not reported	Not reported Agave species (*)
5-methyl-2-furfural (233793) [58]	4.64	Caramellic/spicy [59]	Medium	Process/cooking [60] Process/Geo. Origin (*)
α-terpineol (114833) [61]	8.33	Floral/citrus [62]	Not reported	Agave plant [63] Agave species (*)

## Data Availability

The original contributions presented in the study are included in the article, further inquiries can be directed to the corresponding author.
